# On the Animal Alkaloids

**Published:** 1890-04

**Authors:** 


**On the Animal Alkaloids. The Ptomaines, Leucomaines and Extractives in their Pathological Relations.** By SIR WILLIAM AITKEN, KNT., M. D., L. L. D., F. R. S., Professor of Pathology in the Army Medical School. Second Edition. Pp. 105. Philadelphia: P. Blakiston, Son & Co. 1889.

The important position which the subject of ptomaines and leucomaines has taken in the theory of disease, renders a knowledge of these important substances, and their toxic effect upon the human system, an imperative necessity to the physican of to-day.

Prof. Aitken has given us an excellent little work upon the subject, and it should be carefully read by every physician who wishes to keep abreast of the scientific work and thought of the period.

This little work is terse, concise, and filled with excellent thoughts and suggestions. We most heartily recommend it to the careful perusal of every wide-awake physician. J. A. M.

